# The cold tolerance of the northern root-knot nematode, *Meloidogyne hapla*

**DOI:** 10.1371/journal.pone.0190531

**Published:** 2018-01-02

**Authors:** Xiaojing Wu, Xiaofeng Zhu, Yuanyuan Wang, Xiaoyu Liu, Lijie Chen, Yuxi Duan

**Affiliations:** 1 College of Plant Protection, Shenyang Agricultural University, Shenyang, Liaoning, China; 2 College of Biology science and technology, Shenyang Agricultural University, Shenyang, Liaoning, China; 3 College of Science, Shenyang Agricultural University, Shenyang, Liaoning, China; INRA, FRANCE

## Abstract

The northern root-knot nematode, *Meloidogyne hapla*, is one of the most important nematode pathogens occurring in cold regions. It is a sedentary, biotrophic parasites of plants and overwinter in the soil or in diseased roots. This study showed that the cold tolerance for the second-stage juveniles (J2) of *M*. *hapla* was moderate with the 50% survival temperature (S_50_) of -2.22°C and the fatal temperature was -6°C when cooling at 0.5°C min^-1^. Cryoprotective dehydration significantly enhance cold tolerance of *M*. *hapla* J2 with the lowest S_50_ of -3.28°C after held being at -1°C for 6 h. Moreover, cold shock and cold acclimation had significant effects on the freezing survival of *M*. *hapla* J2. The lethal temperature of eggs was -18°C. Therefore, the cold tolerance of *M*. *hapla* is sufficiently favorable to withstand winters in cold temperature environments.

## Introduction

The northern root-knot nematode, *Meloidogyne hapla*, are sedentary, biotrophic parasites of plants with wide host ranges [1]. The second-stage juvenile (J2) of *M*. *hapla* infect plant roots and induce root-knots, which affect water and nutrient absorption and translocation of root systems. Infection results in reduced crop yield or quality [2], and consequently causes severe economic losses [3, 4]. *M*. *hapla* occurs in cold regions of crop production [4, 5]. According to a previous report, *M*. *hapla* is mainly distributed in cool areas, where the mean temperature is -15°C in the coldest month and approximately 27°C in the warmest month or in high altitude mountainous areas [5]. The only infective stage of *M*. *hapla* is the J2, which must overcome adversely low-temperature environmental conditions before reaching plant roots. It was shown that *M*. *hapla* J2 survived at freezing temperatures [6, 7], and the minimal temperature for development was 8.8°C [8]. Meanwhile, the egg masses also played a key role in overwintering in the soil. *M*. *hapla* eggs survived sub-zero temperatures in the field [9, 10] and developed at the low temperature of 6.74°C [11]. This indicates the ability of *M*. *hapla* to survive at low temperatures.

Cold tolerance is the ability of nematodes to survive low temperatures in their living environment [12]. There are three main cold tolerance strategies in nematodes [13]. Freezing avoidance is where a nematode body fluid remains a liquid below 0°C to avoid freezing. Freezing tolerance enables nematode survival when their bodies undergo ice formation while showing supercooling ability. Cryoprotective dehydration protects nematodes from low temperatures by dehydration caused by surrounding ice. Cold tolerance has been studied in other nematodes, including entomopathogenic nematodes, Antarctic nematodes, and stem nematodes [14–18]. *Steinernema feltiae* and *Heterorhabditis bacteriophora* survived the low temperature of -13°C by cryoprotective dehydration, and the S_50_ of *S*. *feltiae* was -3.73°C [19]. *Panagrellus redivivus* survived at low temperature by freezing tolerance and cryoprotective dehydration [17]. *Marshallagia marshalli* survived rapid exposure to temperature below -30°C [20]. One study determined the cold tolerance of six nematodes with cold acclimation (*Ditylenchus dipsaci*, *P*. *redivivus*, *Steinernema carpocapase*, *Panagrolaimus rigidus*, *Rhabditophanes* sp. and *Panagrolaimus davidi*), while the S_50_ of *P*. *davidi* was -43.6°C lower than the others [12].

Cold acclimation is an adaptive response of organisms to low temperature that increases their capacity to tolerate freezing, and this response has been observed in *P*. *redivivus*, *P*. *davidi* [21, 22], *S*. *feltiae*, *S*. *riobrave*, *S*. *carpocapsae*, *S*. *anomaly* and *H*. *bacteriophora* [18, 23]. In a variety of prokaryotes and eukaryotes, cold shock improved cold tolerance by inducing cold shock proteins [24]. The CspA was a major cold-shock protein induced by *Escherichia coli* when *E*. *coli* was subjected to cold shock [25, 26]. Nematodes *H*. *bacteriophora* and *Trichinella nativa* produced proteins to respond to cold shock [27, 28]. However, the effects of cold shock and cold acclimation on the *M*. *hapla* J2 are unknown, although they can survive at low temperatures.

In this study, we investigated the effect of low temperature on the survival of *M*. *hapla* J2 and the in vitro hatch rate of egg masses. We determined the influence of cryoprotective dehydration, cold acclimation and cold shock, on *M*. *hapla* cold tolerance.

## Methods and materials

### Nematode culture

The *M*. *hapla* were generous gift by Congli Wang (Northeast Institute of Geography and Agroecology, Chinese Academy of Sciences), and maintained on a nematode-susceptible tomato (L-402) in the greenhouse according to the described by Forge and MacGuidwin[6]. The eggs were collected by root bleaching and centrifugation with 36% (wt/vol) sucrose [29, 30]. Eggs were hatched in sterile distilled water at 25°C under dark conditions. J2 were collected 24 h after hatching and used for the experiment.

### Freezing regime

A 50-μl suspension containing approximately 20 *M*. *hapla* J2 nematodes were transferred to a 0.5-ml Eppendorf tube and placed in a cooling block. The temperature of cooling block was controlled by a programmable cooling device (Temperature chamber: TEMI990). The samples were cooled from 1°C to various minimum temperatures (T_min_: -2, -3, -4, -5, -6°C) at 0.5°C min^-1^ and frozen by adding ice crystals (made by AWT) at T_min_ and held for 30 min, then rewarmed to 1°C at 0.5°C min^-1^. The samples were then removed from the cooling block. After thawing, 300 μl AWT was added to the samples, and placed at room temperature for 24 h. Survival was determined by counting the proportion of moving nematodes after a mechanical stimulus by touching nematodes with a homemade eyelash-needle. Control samples were unfrozen at -1°C. Two runs of this regime were used with 5 replicates per run. The temperature at which 50% of the J2 were killed (S_50_) was determined using a Probit analysis [12, 17, 19].

### Cryoprotective dehydration regime

To test whether the cold tolerant mechanism of *M*. *hapla* J2 was a cryoprotective dehydration strategy, samples were cooled from 1°C to -1°C at 0.5°C min^-1^, frozen by inoculating with ice crystals at -1°C and held for a specific period time (2, 6, 12 h) before cooling to T_min_ (-3, -4, -5°C) at 0.5°C min^-1^. They were kept at T_min_ for 30 min and finally rewarmed to 1°C at 0.5°C min^-1^. Two runs of this regime were used with 5 replicates per run. Survival was determined as previously described.

### Cold shock

To test the effect of cold shock on the survival of *M*. *hapla* J2, samples were cooled from 1°C to -1°C at 0.5°C min^-1^ and held for 1 h at -1°C. They were rewarmed to 1°C at 0.5°C min^-1^ in cool block, and then samples were taken from cool block and maintained at room temperature for 1 h [17] before exposed to T_min_ (-3, -4, -5°C) using similar methods as in the ‘freezing regime’. Survival was detected as before.

### Cold acclimation

To test the effect of cold acclimation on survival of *M*. *hapla* J2, samples were acclimated at 4°C for 12 h before cooling to T_min_ (-3, -4, -5°C) at 0.5°C min^-1^, the cold exposure was using the ‘freezing regime’. Survival was detected as before.

### Effect of low-temperature on egg mass hatching rates

To test the hatch rate of the egg mass of *M*. *hapla*, which was exposed to low temperature, seven temperature treatments (T_min_: -2, -6, -10, -14, -15, -16, and -18°C) were used. Similar sizes of fresh egg masses were chosen and sterilized by 0.4% NaOCl solution, then placed in the homemade hatching pond. Samples cooled from 1°C to T_min_ at 0.5°C min^-1^, kept at T_min_ for 30 min and then warmed to 1°C at 0.5°C min^-1^, finally the samples were removed from the cooling block. Control samples were kept at 25°C. All treatments were hatched in sterile distilled water at 25°C. Each treatment had 3 replicates per run, and two runs of this regime were used. The number of eggs in each egg-mass were 672 ± 5.5 (mean ± SE) on average. After 10 days, the number of hatching nematodes compared to the proportion of eggs were examined according to the formula as below:

HatchingPercentage(%)=(thenumberofhatchingJ2/thenumberofeggsineggmass)×100

### Statistical analysis

All statistical analyses were calculated by using SPSS v. 17.0 [31]. Probit analysis models were used to determine the temperature at which 50% of nematodes were killed (S_50_). The minimum temperatures (T_min_) were log_10_ transformed to linearize the data. The relative median potency (RMP) estimated the difference of the S_50_ between two groups. Significant differences were defined between groups, if the 95% confidence limits (CL) of RMP estimation does not encompass the value 1. The effect of treatments on survival were tested using a factorial ANOVA [12, 17, 19].

## Results

### Freezing regime and cryoprotective dehydration

**Fig 1:**
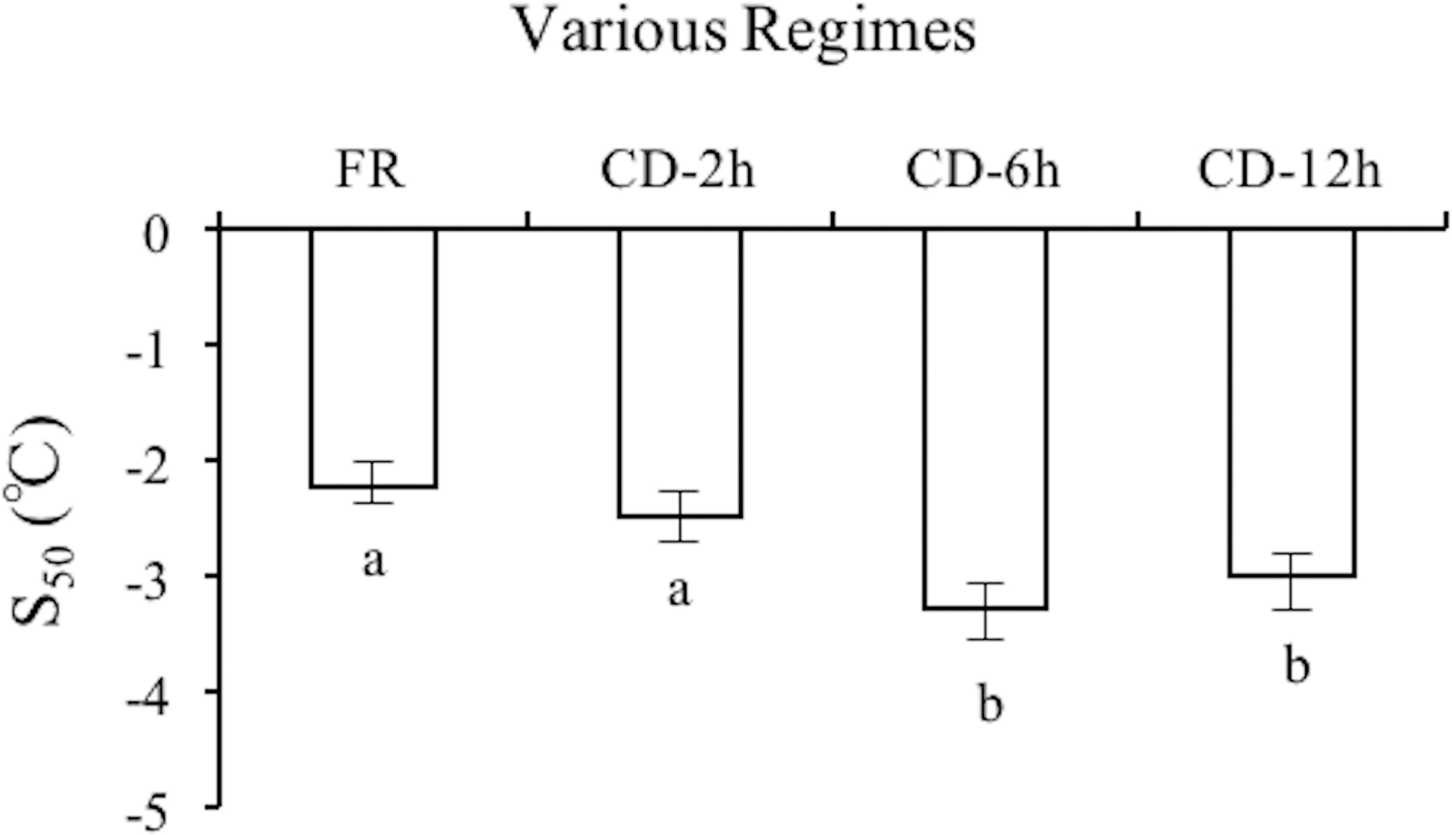
The S_50_ values of *M*. *hapla* J2 exposed to freezing regime and cryoprotective dehydration. FR = freezing regime, CD-2 h = Cryoprotective dehydration regime that held for 2 h at -1°C before cooled to T_min_, CD-6 h = Cryoprotective dehydration regime that held for 6 h at -1°C before cooled to T_min_, CD-12 h = Cryoprotective dehydration regime that held for 12 h at -1°C before cooled to T_min_. The bars are the estimations in the 95% confidence limits. The different lowercase letters on the bars represent significantly different among various regimes, according to RMP estimates. N = 10.

The effect of temperature on survival of *M*. *hapla* J2 in the freezing regime is significant. With the temperature reduced, survival decreased significantly (Welch test, Alpha = 0.05, df = 3; *P* < 0.001) (S1 Fig). The S_50_ values of freezing regime was -2.22°C (95%CL = -2.01, -2.38°C). Moreover, the S_50_ values were significantly decreased with an increased freezing time at -1°C after 2–6 h, which was lower 1.06°C after 6 h compared to the freezing regime (RMP = 1.47; 95%CL = 1.23, 2.10), but there was no significant difference between 6 h and 12 h (Fig 1).

## Cold shock and cold acclimation

**Fig 2:**
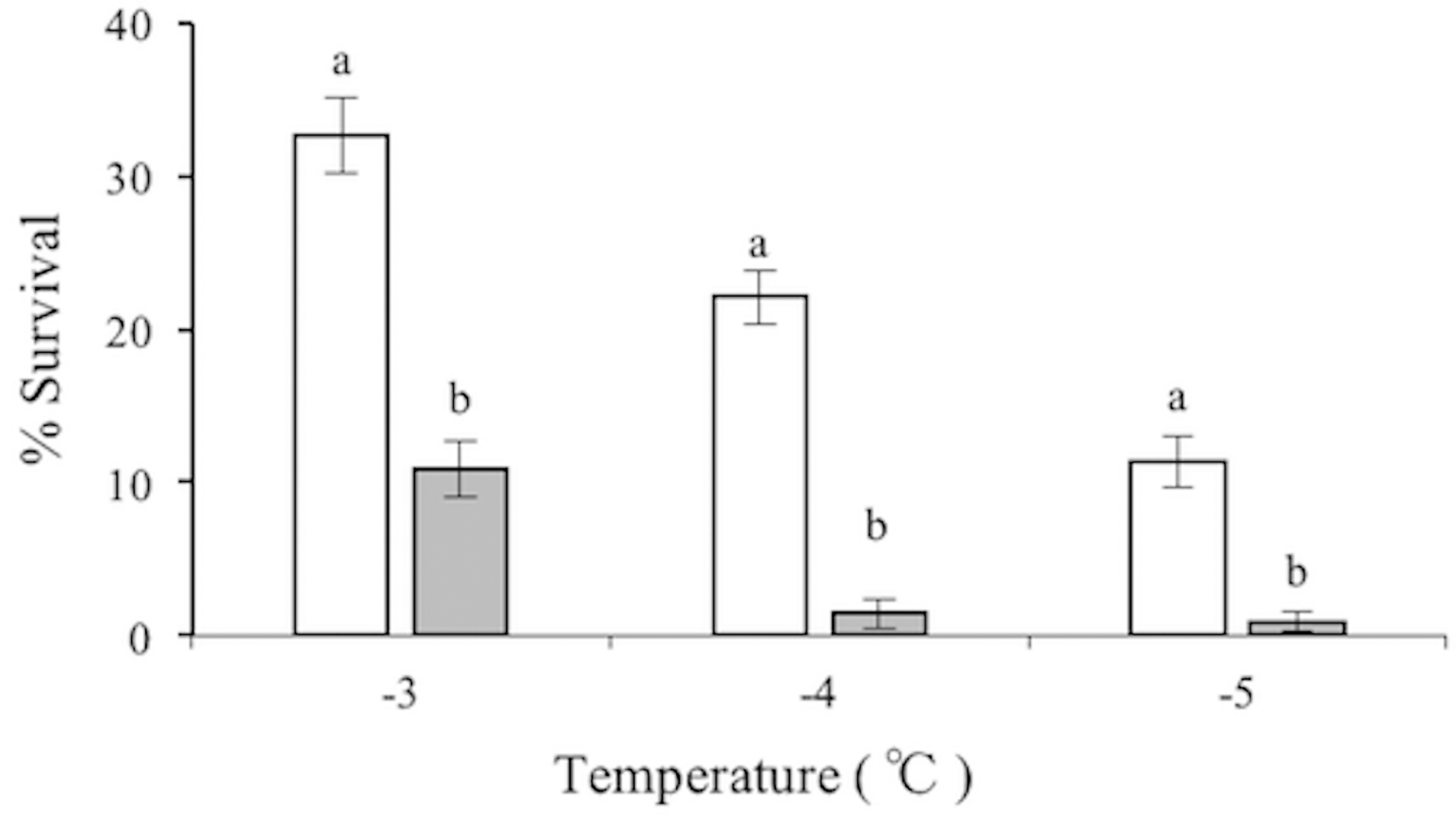
The effect of cold acclimation on survival of *M*. *hapla* J2. Samples acclimated at 4°C (open bars), and survival at freezing regime without acclimation (filled bars). The different lowercase letters on the bars represent significantly different (*P* < 0.05) between treatments at the same temperature. The bars are the mean ± SE in this figure. N = 10.

**Fig 3:**
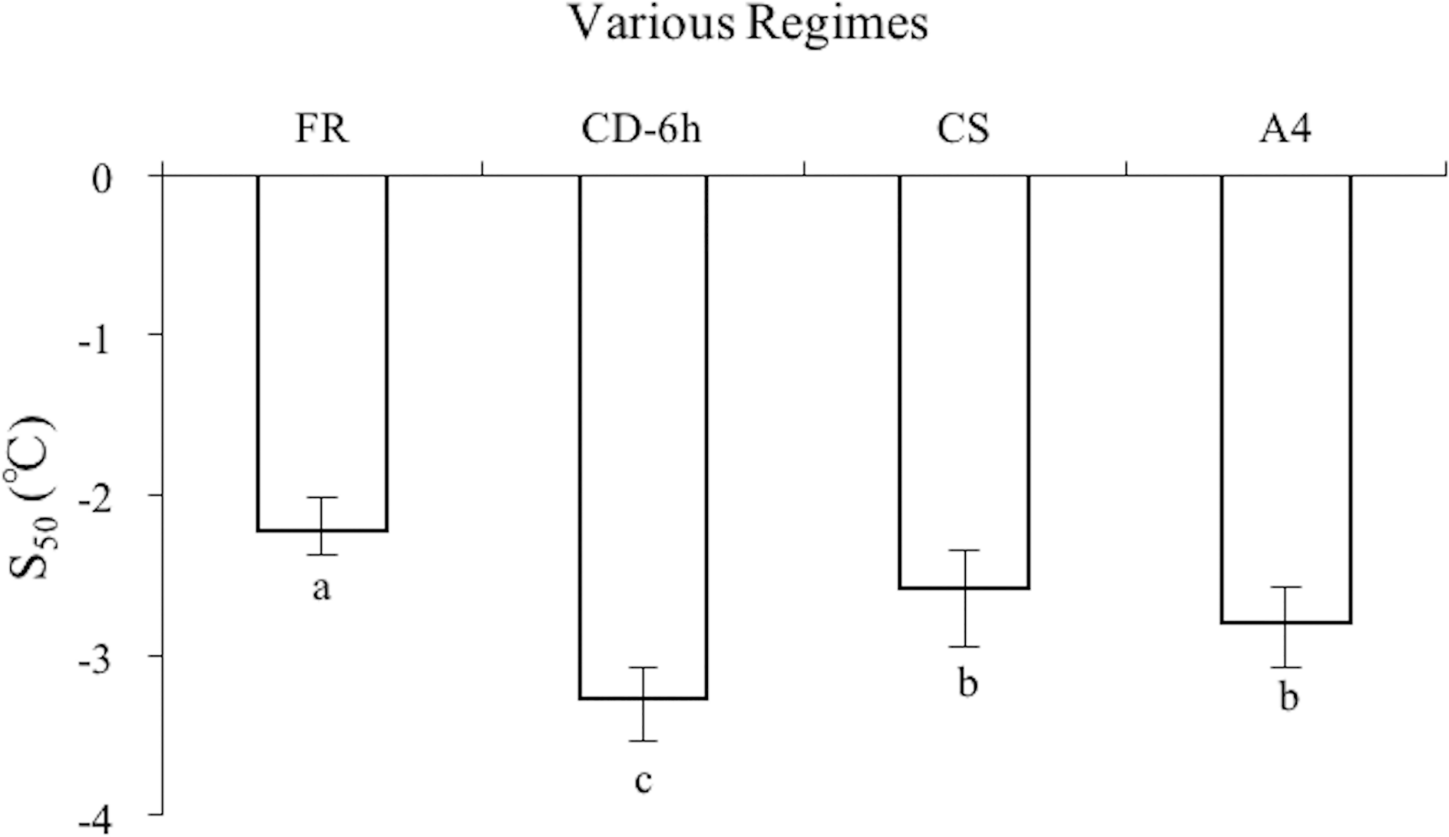
The S_50_ values of *M*. *hapla* J2 subjected to various regimes. FR = freezing regime; CD-6 h = Cryoprotective dehydration regime that held for 6 h at -1°C before cooled to T_min_, CS = cold shock at -1°C for 1 h and then kept at room temperature for 1 h before cooled to T_min_, A4 = acclimated at 4°C for 12 h before cooled to T_min_. The bars are the estimations in the 95% confidence limits. The different lowercase letters on the bars represent significantly different among various regimes, according to RMP estimates. N = 10.

Survival of *M*. *hapla* J2 subjected to cold shock at -1°C for 1 h was significantly increased (S2 Fig). The S_50_ was -2.58°C (95%CL = -2.34, -2.95°C), lower than the S_50_ in the freezing regime (RMP = 1.16; 95%CL = 1.05, 1.32). Survival of *M*. *hapla* J2 significantly improved by acclimated at 4°C compared to the freezing regime (Fig 2). And that the S_50_ was -2.79°C (95%CL = -2.58, -3.08°C), which was significantly different from the freezing regime (RMP = 1.26; 95%CL = 1,12, 1,52). Moreover, the S_50_ values of various regimes were compared in Fig 3. The S_50_ of cryoprotective dehydration for 6 h was lower than those achieved through cold shock (RMP = 1.27; 95%CL = 1,07, 1,75) and acclimation (RMP = 1.17; 95%CL = 1,04, 1,46), but there were no significant differences between cold shock and acclimation.

### Effect of low-temperature on egg mass hatching percentage

**Fig 4:**
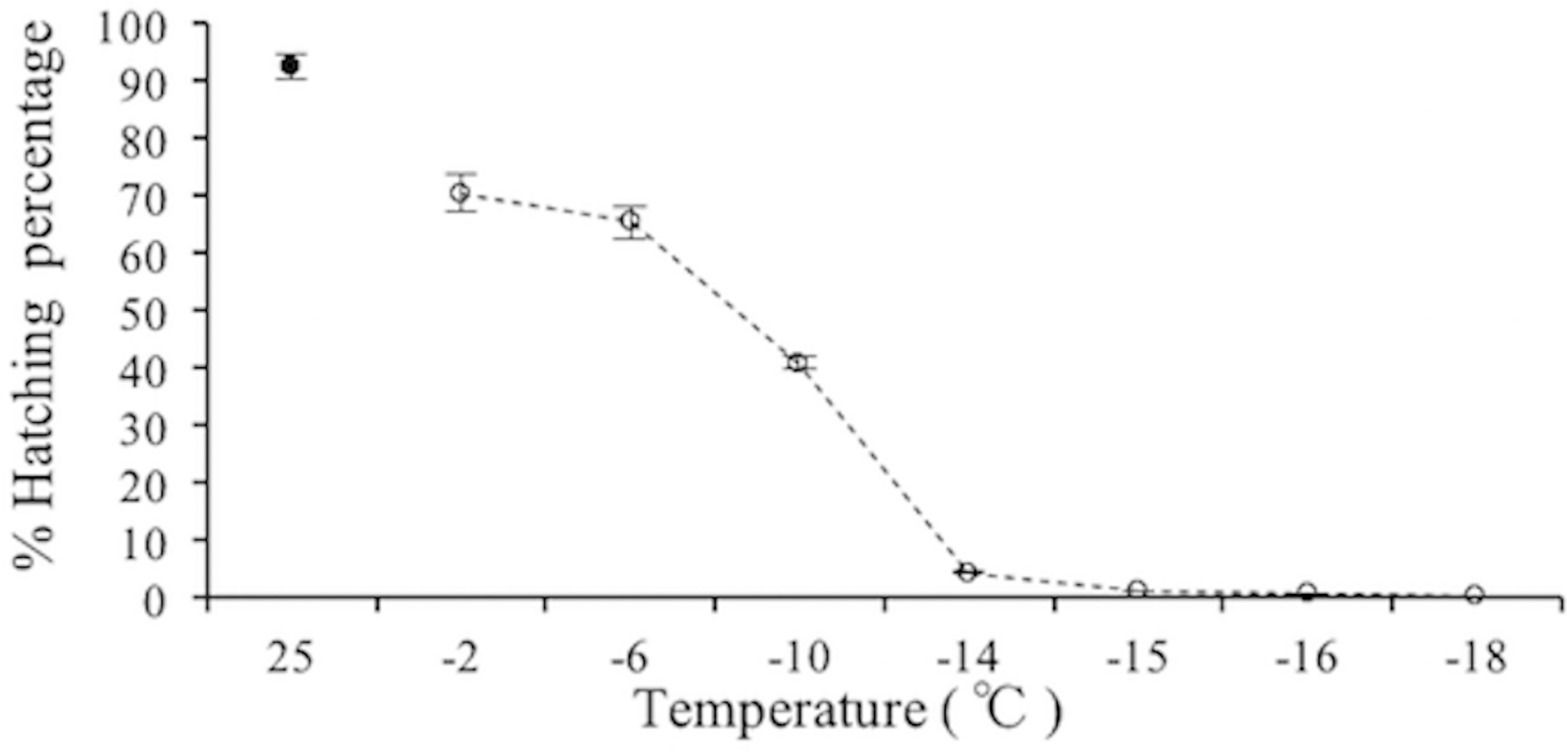
The hatching percentage for egg mass of *M*. *hapla* at various low temperature. Open circles = hatching percentage at various low temperature during 10 days, Closed circle = 25°C. The values are the mean ± SE in this figure. N = 6.

The hatching percentage for egg mass of *M*. *hapla* declined with decreasing temperatures (Welch test, Alpha = 0.05, df = 6; *P* < 0.001) (Fig 4).

## Discussion

*M*. *hapla* J2 indeed survived at low temperature. Previous studies showed that the *M*. *hapla* J2 were frozen spontaneously at -8°C at the freezing rate of 1°C min^-1^ [7]. However, in our experiment, the freezing rate was 0.5°C min^-1^ because the physical damage of ice formation decreased using a slow freezing rate [32]. The results indicated the cold tolerance of *M*. *hapla* J2 was modest, with the S_50_ of -2.22°C and fatal temperature of -6°C in the freezing regime. Comparatively, the S_50_ of *P*. *davidi* was -43.6°C [12].

Inoculating ice could make the surrounding medium freeze rapidly at high subzero temperatures and provide the basic environment of cryoprotective dehydration of nematodes [33]. Cryoprotective dehydration [13, 33–36] had a significant effect on cold tolerance of some nematodes, such as *P*. *davidi*, *S*. *feltiae*, and *H*. *bacteriophora* [16, 19]. In our freezing regime, the S_50_ of *M*. *hapla* J2 was -2.22°C, and the value significantly decreased with increased time at -1°C in the cryoprotective dehydration regime. This result might explain that cryoprotective dehydration had an effect on the cold tolerance of *M*. *hapla* J2. Moreover, a previous study showed that following exposure to -15°C for 10 min, the cuticle of *M*. *incognita* J2 had been torn away from the body by freezing but not in the *M*. *hapla* J2 [7]. Thus, the cold tolerance of this species may have been aided by cryoprotective dehydration and freezing tolerance.

Survival of *M*. *hapla* J2 was improved significantly by cold shock at -1°C for 1 h, and the S_50_ was -2.58°C, which was lower than the freezing regime. Cold shock occurs in a variety of organisms [24]. *H*. *bacteriophora* induces the trehalose-6-phosphate synthase by cold shock [27], and Hsp70 was markedly increased to in response to cold shock in *Trichinella native* [28], while, cold shock had no significant effect on survival of *P*. *redivivus* [17] and *S*. *feltiae* [19].

*M*. *hapla* J2 that were acclimated at 4°C for 12 h showed a significant enhancement in survival. These were similar results to those found by Forge and MacGuidwin [6]. Cold acclimation response has been studied on a variety of nematode species [33]. The supercooling points of *P*. *redivivus* were decreased by cold acclimation, enabling survival at lower temperatures [21]. The freezing tolerance of *S*. *feltiae*, *S*. *anomaly* and *H*. *bacteriophora* was increased after cold acclimation [18]. Moreover, cold acclimation response induced trehalose accumulation in entomopathogenic nematodes (*S*. *feltiae*, *S*. *riobrave*, *S*. *carpocapsae*) [23] and *P*. *davidi* [22]. Meanwhile, calcium or calmodulin-mediated signaling played a pivotal role in response to cold acclimation in plants [37]. However, the mechanism of cold acclimation and cold shock effects on the survival of *M*. *hapla* J2 needs to be further investigated.

In nature, nematodes usually overwinter by egg masses in the plant debris or soil when the soil temperature is subzero. In this study, the percentage of egg-hatching for *M*. *hapla* within 10 days was 65.30% at -6°C and 4.27% at -14°C, indicating that *M*. *hapla* survived at low temperatures, which was similar to the results by Daulton et al. [9]. However, pre-exposure to 4, 12 and 18°C for two weeks did not significantly affect the hatch rate of the *M*. *hapla* egg masses [7].

We found that the lethal temperature of *M*. *hapla* J2 was -6°C with freezing by adding ice, and cryoprotective dehydration improved the cold tolerance of *M*. *hapla* J2. The lethal temperature of *M*. *hapla* egg mass in our experiments was -18°C after freezing spontaneously. Moreover, cold acclimation and cold shock significantly improved the cold tolerance of *M*. *hapla* J2, which is advantageous for withstanding the winter in cold environments.

## Supporting information

S1 Fig Survival of *M*. *hapla* J2 after cooling to various minimum temperatures. Treatments were frozen by adding ice (open circle), and unfrozen (closed circle). The values are the mean ± SE in this figure. N = 10.
(TIFF)

S2 Fig The effect of cold shock on survival of *M*. *hapla* J2. Samples cold shocked at -1°C for 1 h and then kept at room temperature for 1 h before being cooled to T_min_ (filled circles), and survival at freezing regime at the corresponding test temperature (-3, -4, -5°C) without cold shock (open circles). The values are the mean ± SE in this figure. N = 10.
(TIFF)
