# Supplementary figures and images for: The cold tolerance of the northern root-knot nematode, Meloidogyne hapla
Source: PLoS One. 2018 Jan 2;13(1):e0190531. doi: 10.1371/journal.pone.0190531 (PMC5749844; doi:10.1371/journal.pone.0190531)

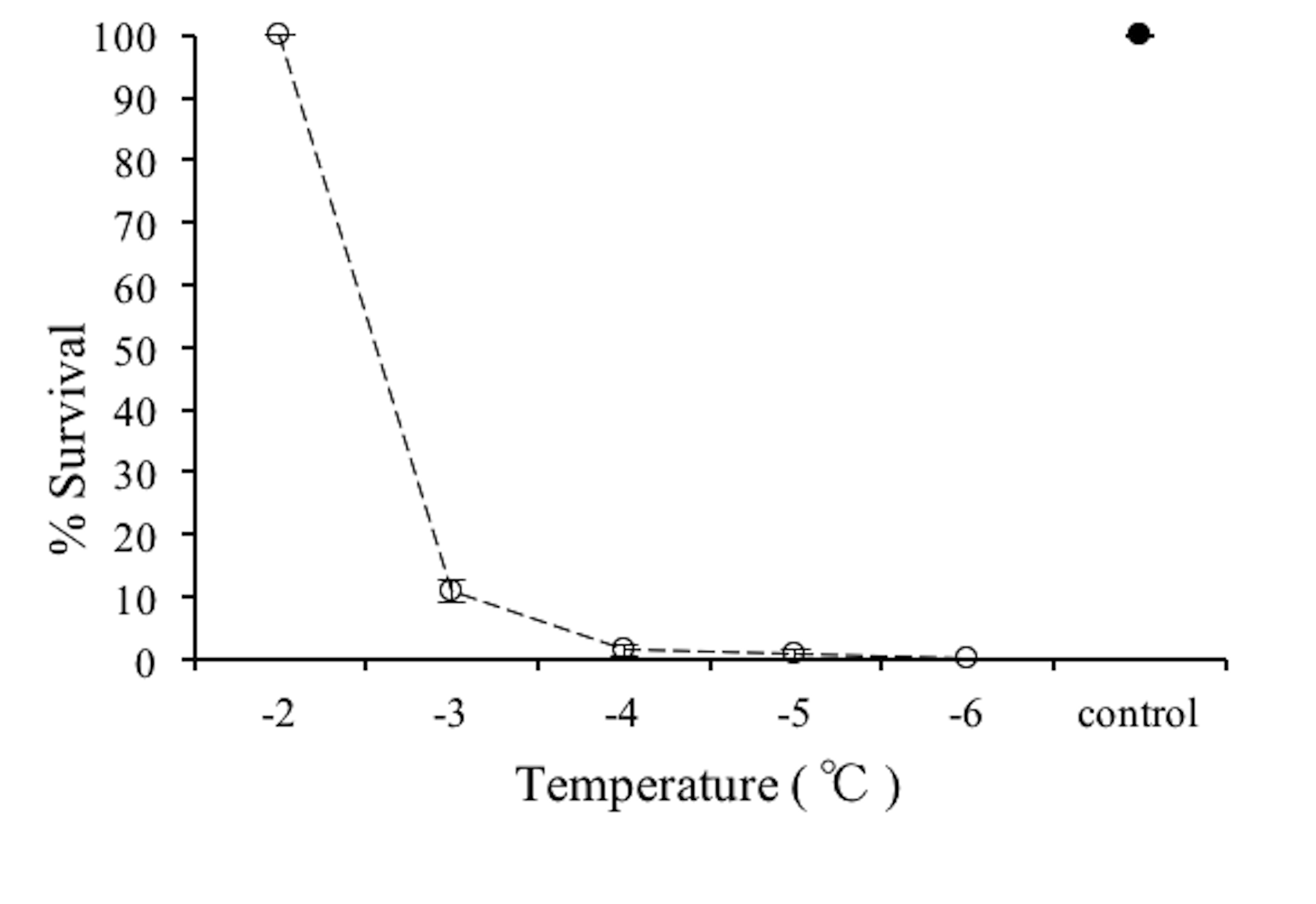

Supplement: S1 Fig — Treatments were frozen by adding ice (open circle), and unfrozen (closed circle). The values are the mean ± SE in this figure. N = 10. (TIFF) [file pone.0190531.s001.tiff]

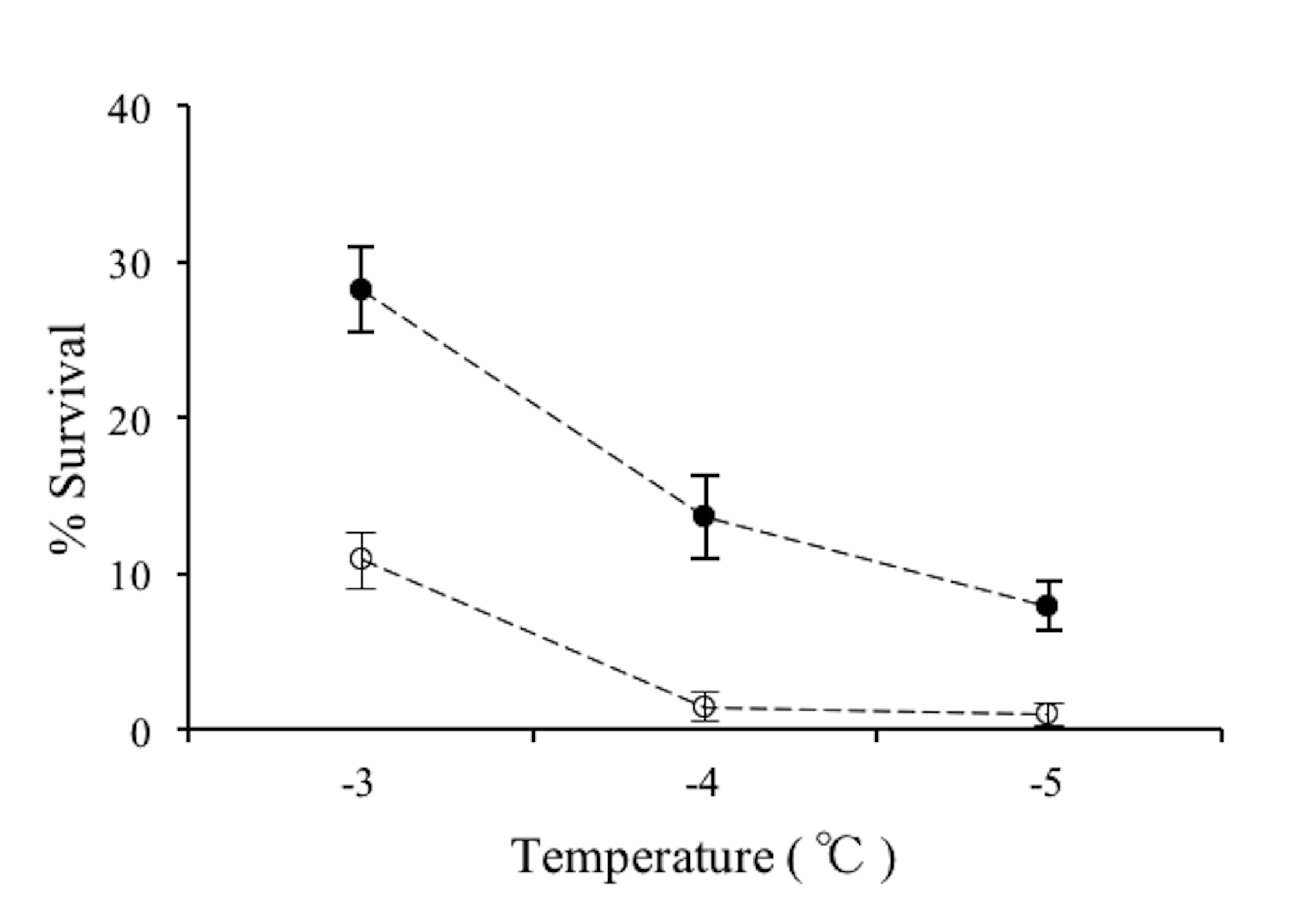

Supplement: S2 Fig — Samples cold shocked at -1°C for 1 h and then kept at room temperature for 1 h before being cooled to Tmin (filled circles), and survival at freezing regime at the corresponding test temperature (-3, -4, -5°C) without cold shock (open circles). The values are the mean ± SE in this figure. N = 10. (TIFF) [file pone.0190531.s002.tiff]
